# Supplementary figures and images for: Mathematical modeling unveils the timeline of CAR-T cell therapy and macrophage-mediated cytokine release syndrome
Source: PLoS Comput Biol. 2025 Apr 9;21(4):e1012908. doi: 10.1371/journal.pcbi.1012908 (PMC11981663; doi:10.1371/journal.pcbi.1012908)

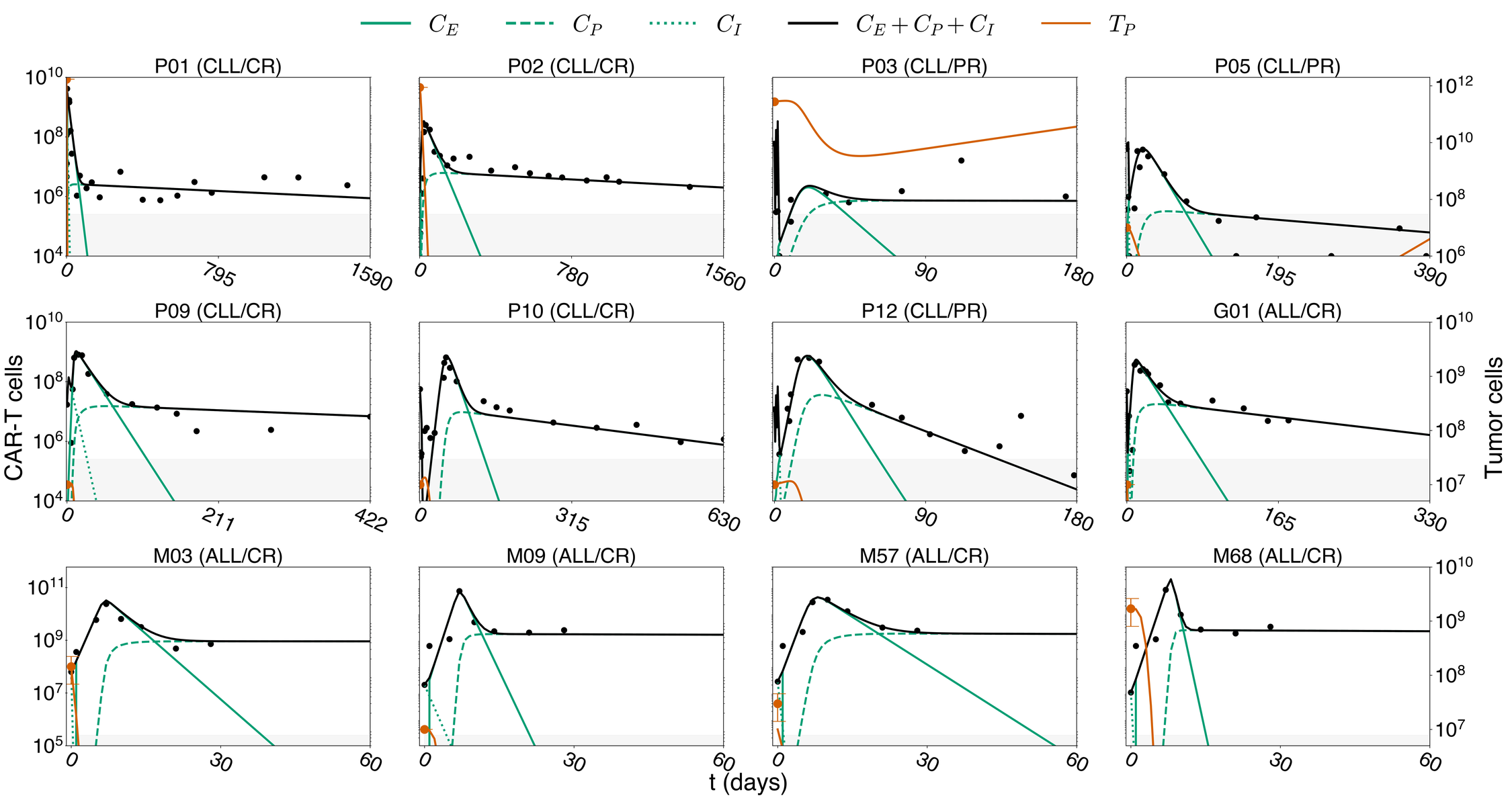

Supplement: S1 Fig — Model fits for selected patients that showed either complete response (CR) or partial response (PR). CR is achieved when the tumor is clinically undetectable, while PR is defined by a final tumor burden at least less than 50% of the initial burden. The CAR-T cell detection threshold of 2.5×10^5^ cells is represented by the gray shaded area. (TIF) [file pcbi.1012908.s001.tif]

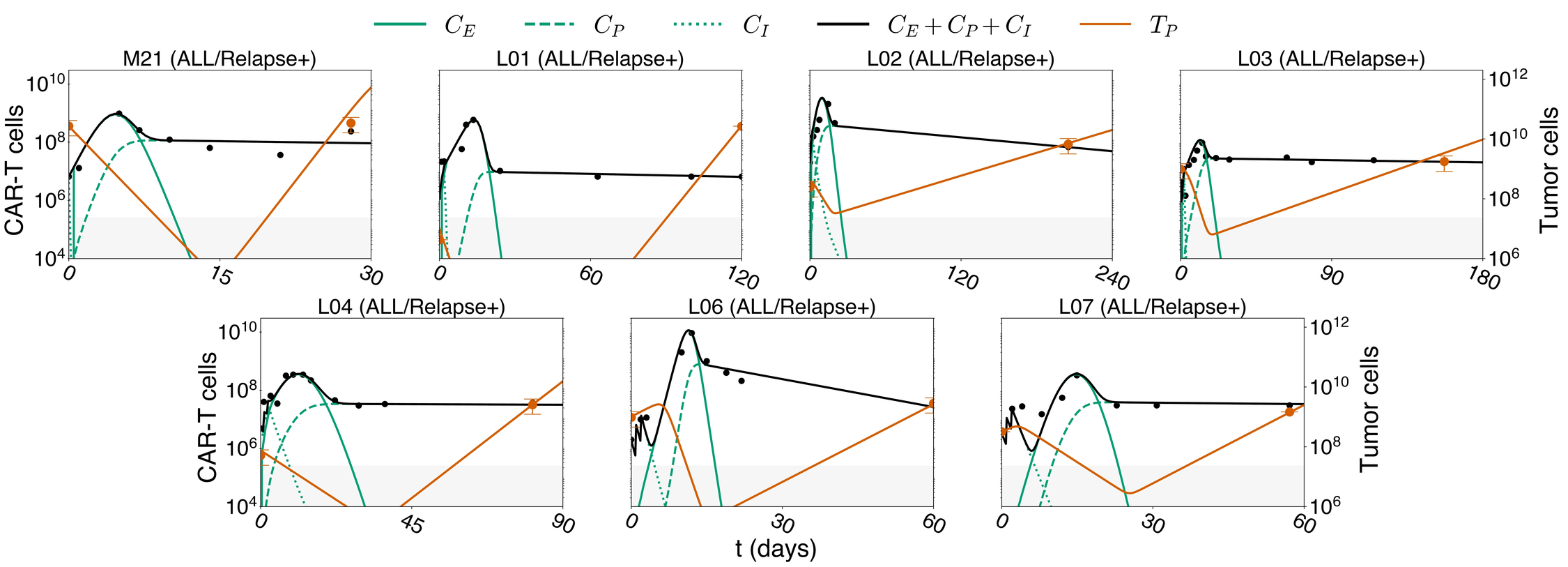

Supplement: S2 Fig — Model fits for selected patients that showed relapse of antigen-positive tumor cells, excluding data points where %blasts approached zero. Tumor cell error bars represent the range of WBCs used in scaling (see Methods). The CAR-T cell detection threshold of 2.5×10^5^ cells is represented by the gray shaded area. (TIF) [file pcbi.1012908.s002.tif]

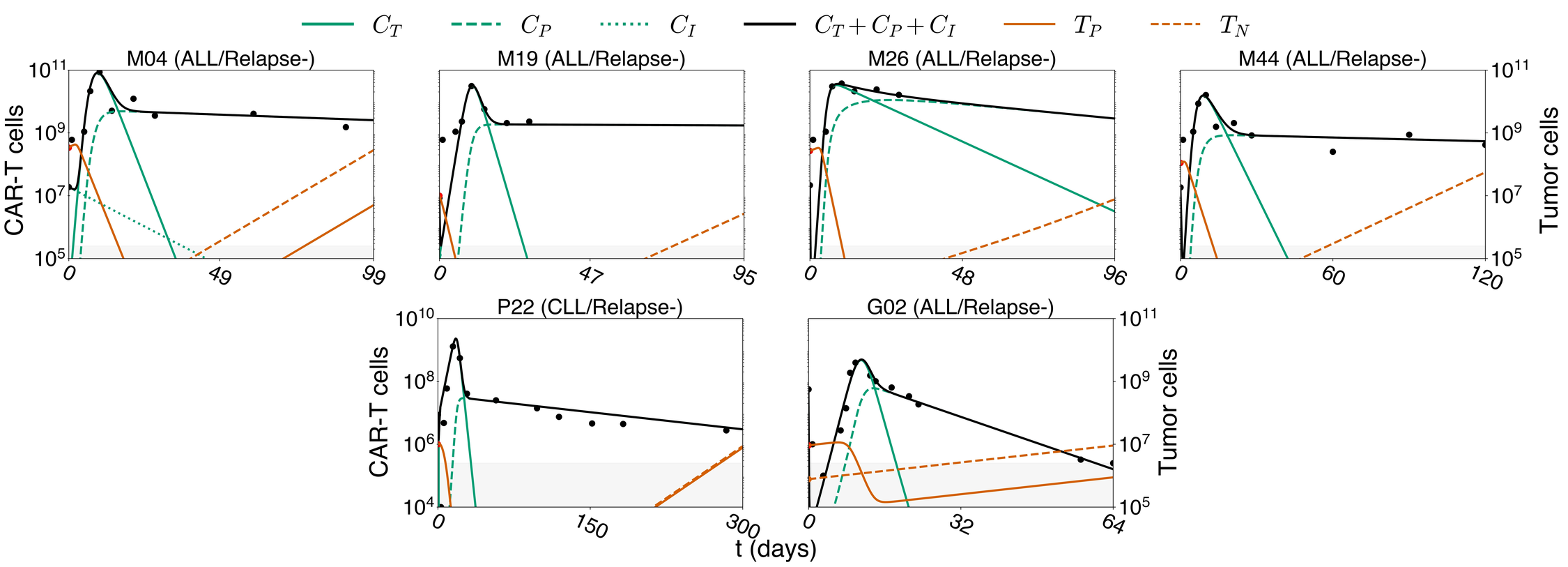

Supplement: S3 Fig — Model fits for selected patients that showed relapse of antigen-negative tumor cells. Except for patient G02, the initial tumor burden (TN(0)) was fitted below the detection limit of 2.5×10^5^ cells (gray shaded area). (TIF) [file pcbi.1012908.s003.tif]

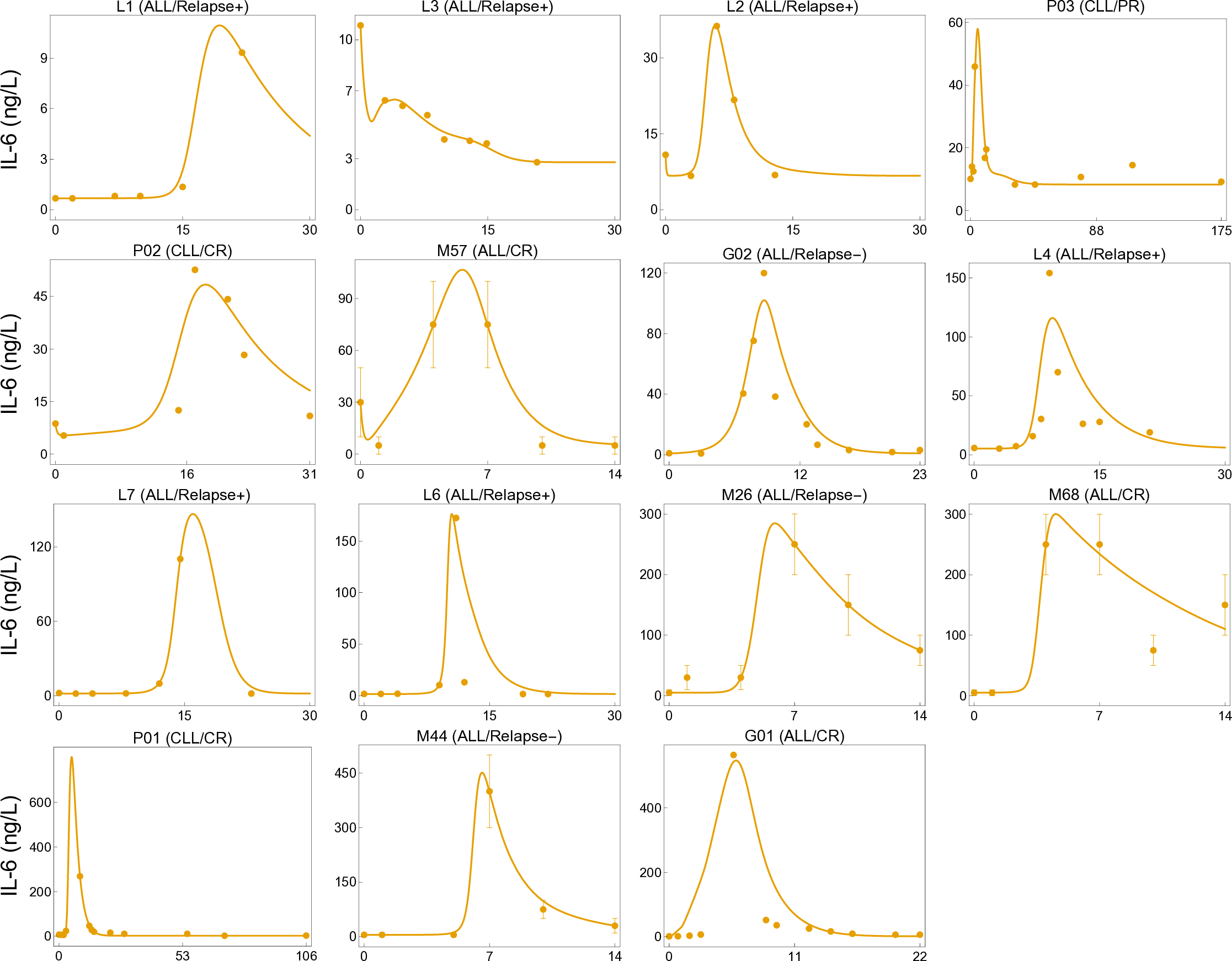

Supplement: S4 Fig — Experimental data points (orange dots) from [1,32,55,57] are compared with model predictions (orange line). When data was presented data as serum fold change, we establish a direct relationship by considering either a baseline value of 1 ng/L [1] or the specific baseline values provided for each patient [56,57]. The mean values within the reported range for dataset [32] are visually represented by bars in the figure. (TIF) [file pcbi.1012908.s004.tif]

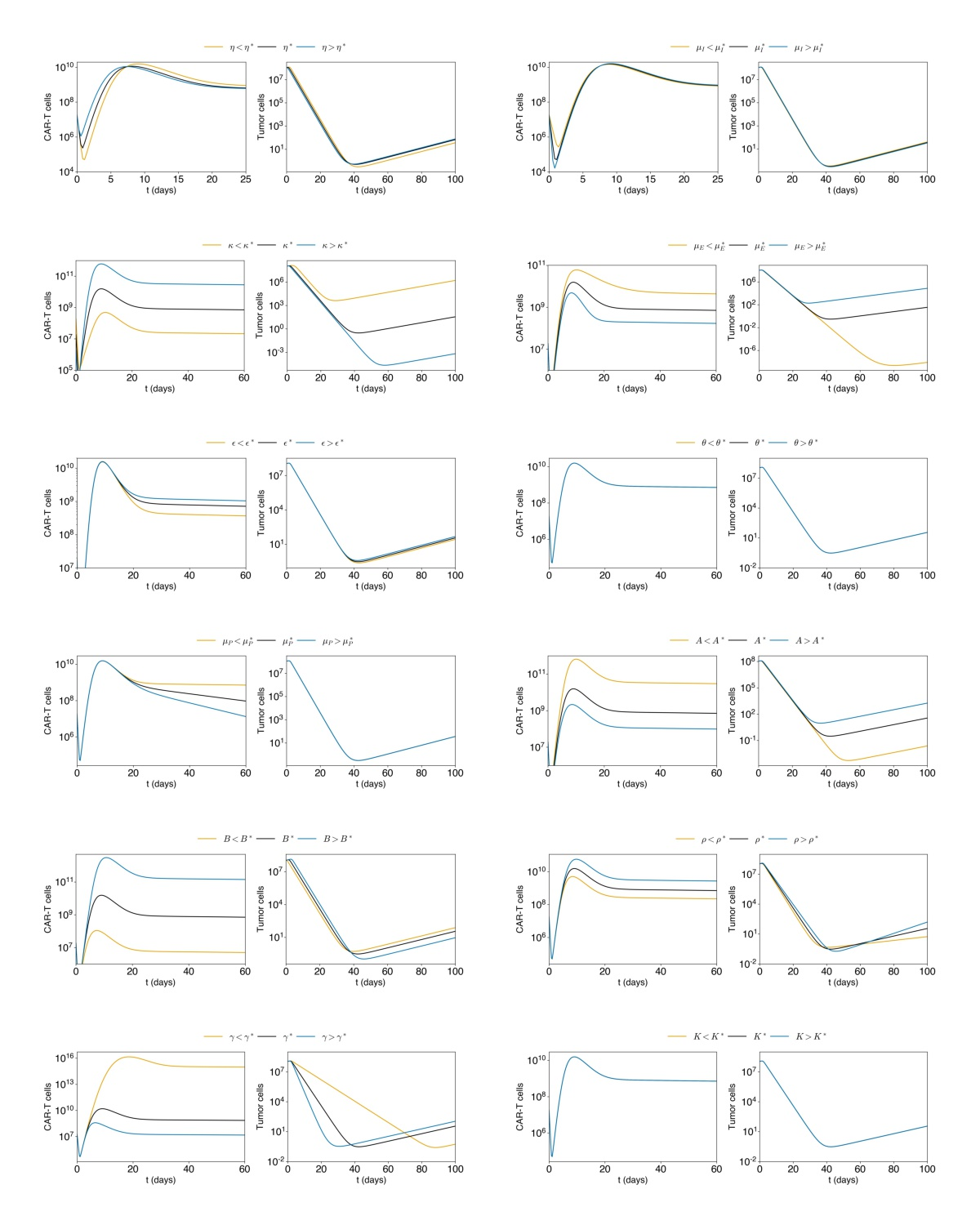

Supplement: S5 Fig — A systematic sensitivity analysis identifies the effect of each of the ten basic mechanistic parameters on the dynamics of CAR-T and tumor cells. The reference simulation is shown in black and alternative scenarios are shown in blue and yellow, where one parameter is changed at a time. (TIF) [file pcbi.1012908.s005.tif]

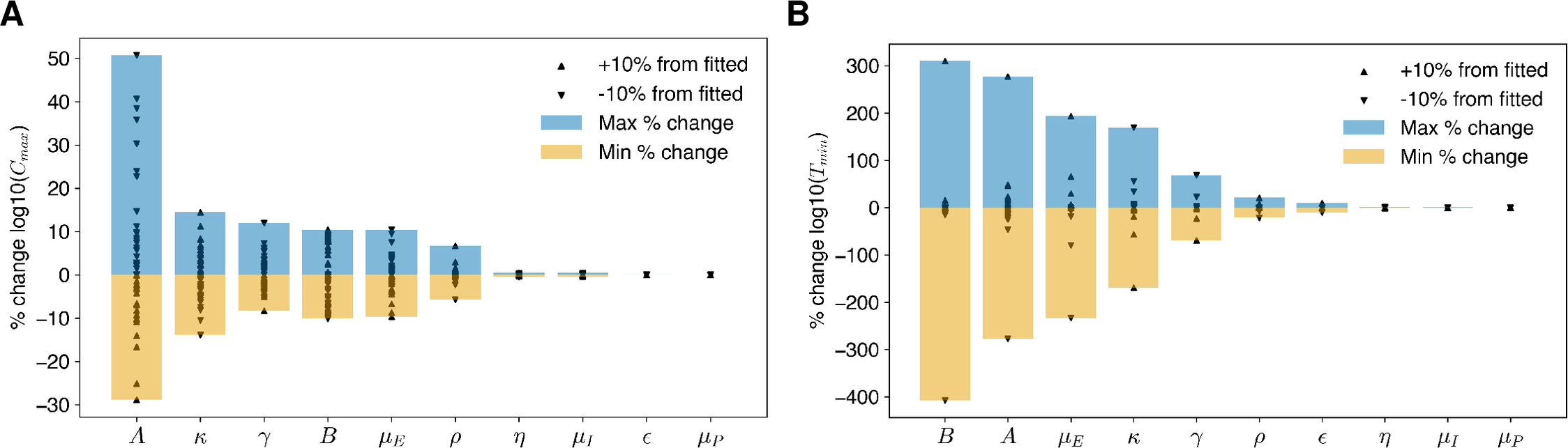

Supplement: S6 Fig — The relative change in A number of CAR-T cells at peak (Cmax) (all patients) and B minimum tumor load (non-responders) achieved during the shrinkage phase (Tmin) were calculated as each parameter varied ± 10% at a time. Yellow and blue bars indicate the maximum and minimum percentage changes, respectively. Upward triangles indicate the % change for a 10% increase, while downward triangles indicate the % change for a 10% decrease in each parameter value. (TIF) [file pcbi.1012908.s006.tif]

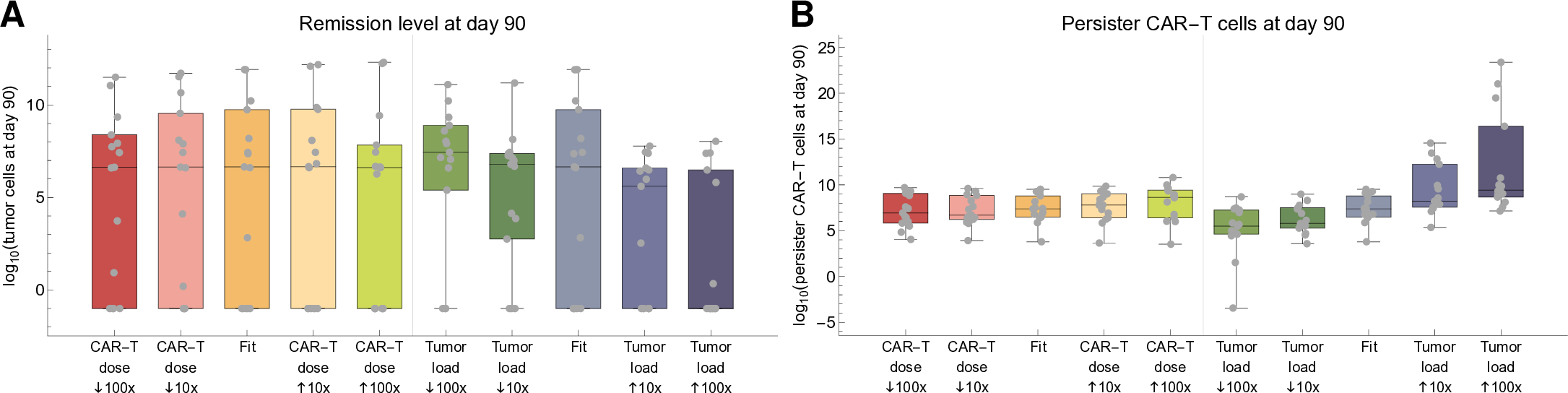

Supplement: S7 Fig — Comparing the standard scenario (Fit) with simulations starting with either a different CAR-T dose or initial tumor burden (10x and 100x smaller and higher). Assessed outcomes: A number of tumor cells at day 90, B number of persister CAR-T cells at day 90. (TIF) [file pcbi.1012908.s007.tif]

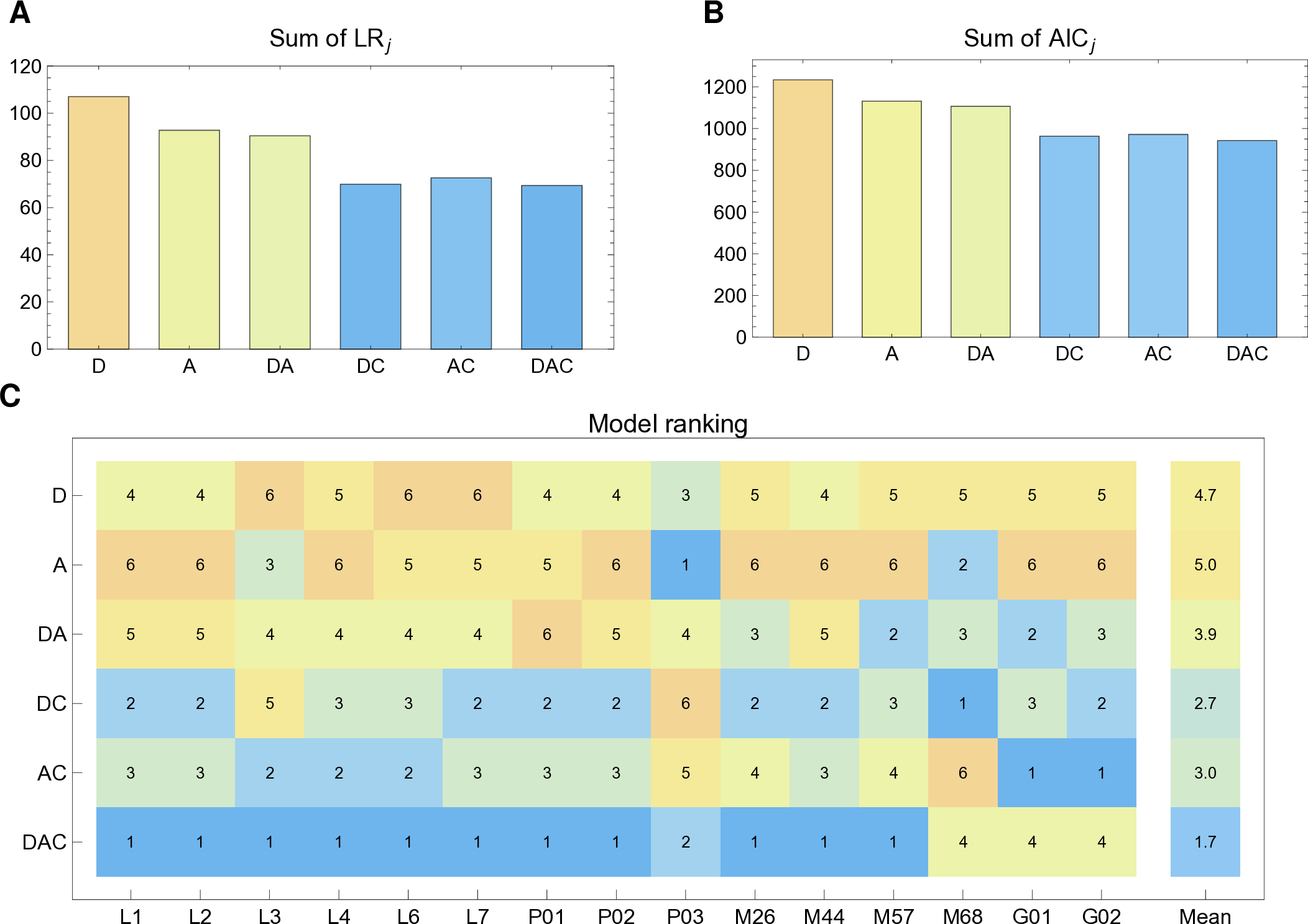

Supplement: S8 Fig — The automated approach used to estimate macrophage and IL-6 parameters in the model considering three activation mechanisms (denoted DAC - Damps, Antigen, CD40) was applied to the reduced models considering 2 or 1 activation mechanisms (DA, DC, AC, D, A), obtained by setting some βi=0. Model C was not considered because CD40 activation depends on the presence of previously activated macrophages. Panels A and B show for each model the sum of LRj (Eq(14)) and AICj (Eq(15)) over all patients. Panel c shows the model ranking among patients. (TIF) [file pcbi.1012908.s008.tif]

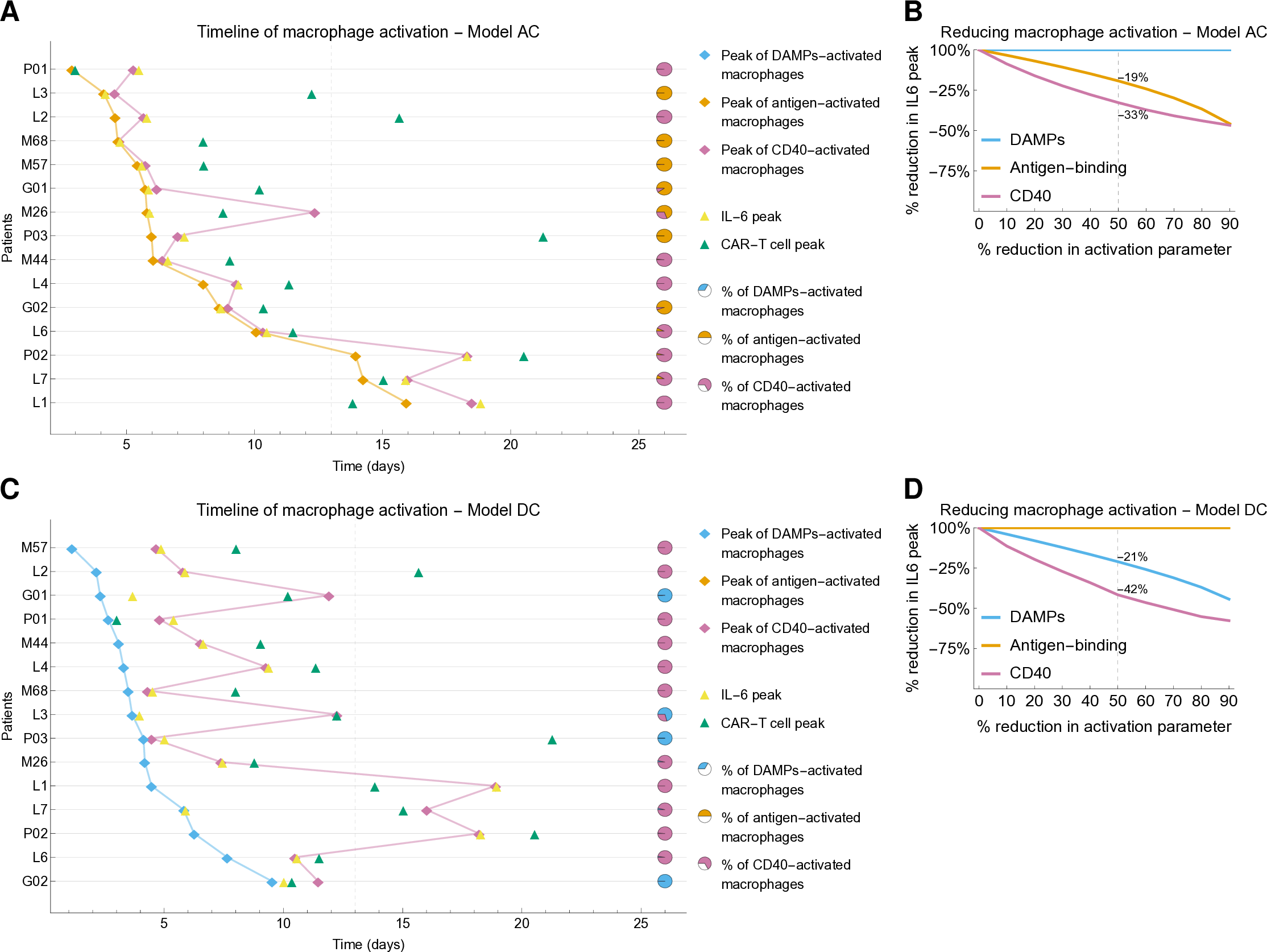

Supplement: S9 Fig — Timelines of macrophage-activation, IL-6 and CAR-T cell peaks for each patient. The percentage of CD40-activated macrophages when all patients are combined was 55% and 75% in models AC and DC, respectively. B,D Simulation results of interventions reducing mechanisms of macrophage activation one at a time, showing the the overall reduction in IL-6 peak. See Figs 7 and 8 for further details. (TIF) [file pcbi.1012908.s009.tif]

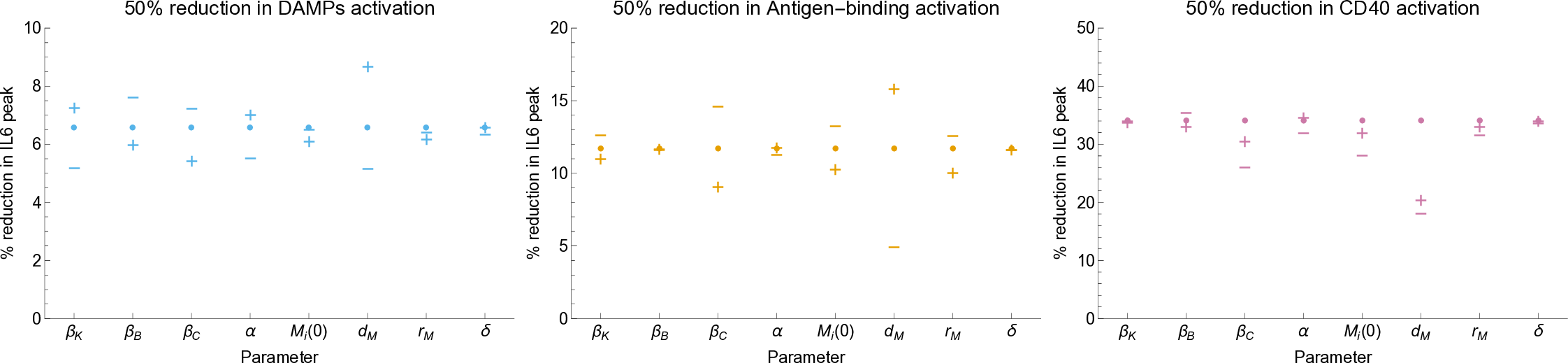

Supplement: S10 Fig — A local sensitivity analysis was performed to assess the effect of varying parameters on the % reduction in IL6 peaks. For each patient, a constant 50% reduction in each activation mechanism was simulated and the mean reduction in IL6 peak was calculated (reference scenario, black dots, same values as shown in Fig 8A fourth panel); this analysis was then repeated by increasing ( + ) and decreasing (–) each parameter in 50% increments. (TIF) [file pcbi.1012908.s010.tif]

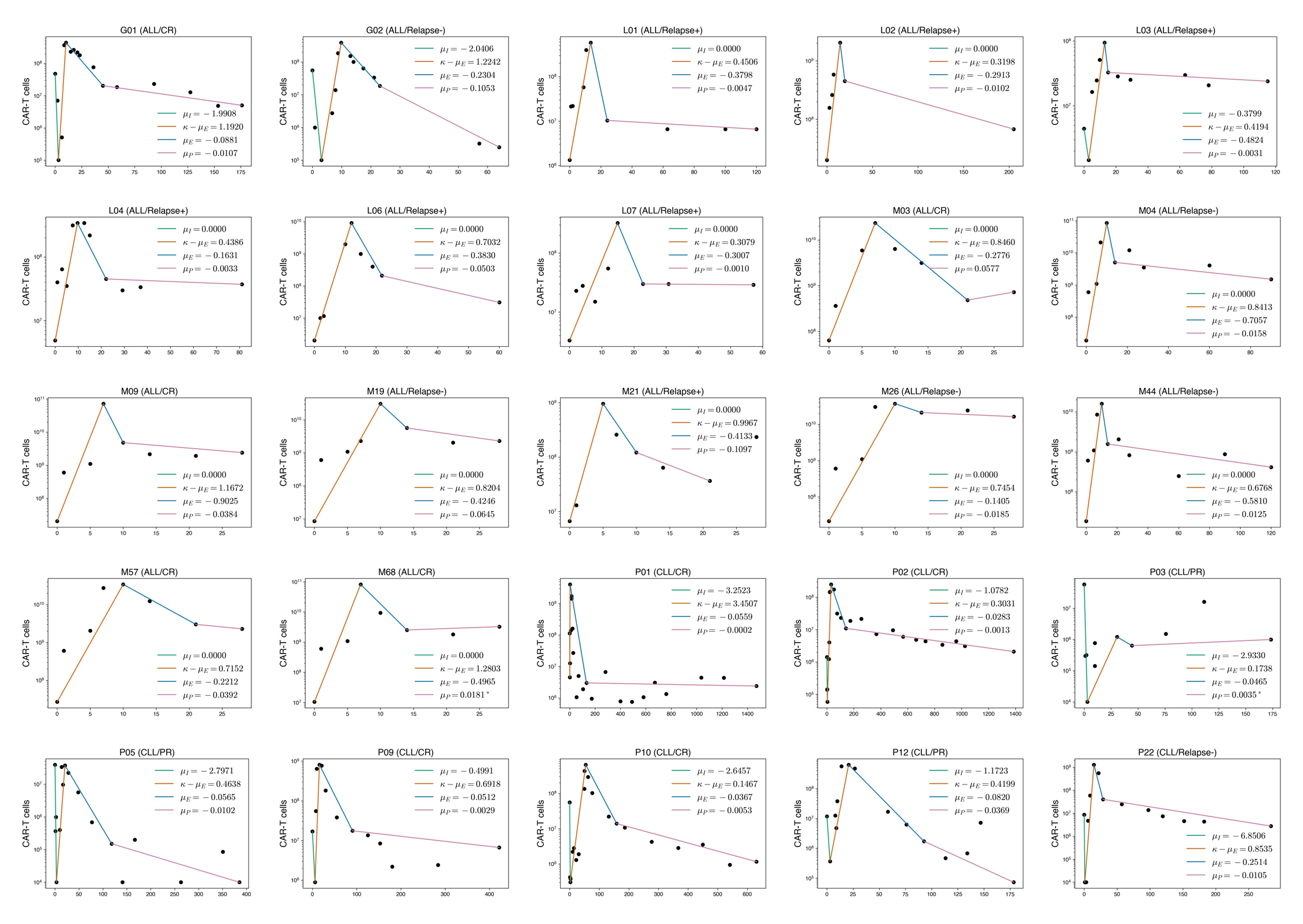

Supplement: S11 Fig — Segmentation of CAR-T multiphasic dynamics was performed by defining an exponential curve for each phase. For the distribution phase, we consider data points ranging from the dose until the minimum level of CAR-T cells observed, until 5 days. For the expansion and contraction phases, we consider all data points within the endpoints, which mark the distribution and persistence phases. If the persistence phase is not well marked, we choose the slope that best describes the interior points. Finally, the distribution phase is defined until the last observed data point. (TIF) [file pcbi.1012908.s011.tif]

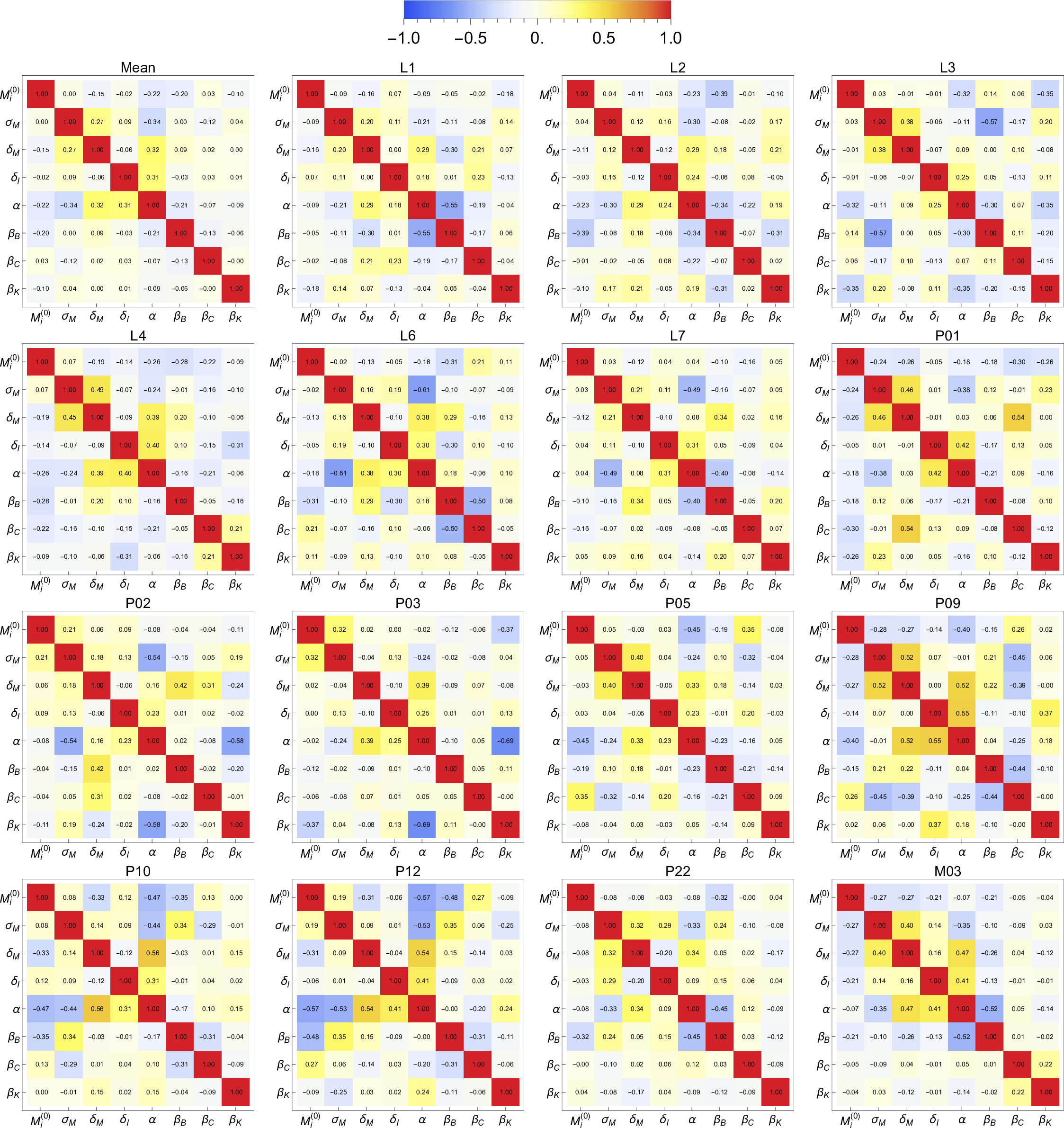

Supplement: S12 Fig — Each plot shows the pairwise correlations between all parameter pairs, considering the 100 best fits obtained after parameter fitting for the IL-6 model, see Methods for details. The median of all pairwise correlations is also shown. (TIF) [file pcbi.1012908.s012.tif]

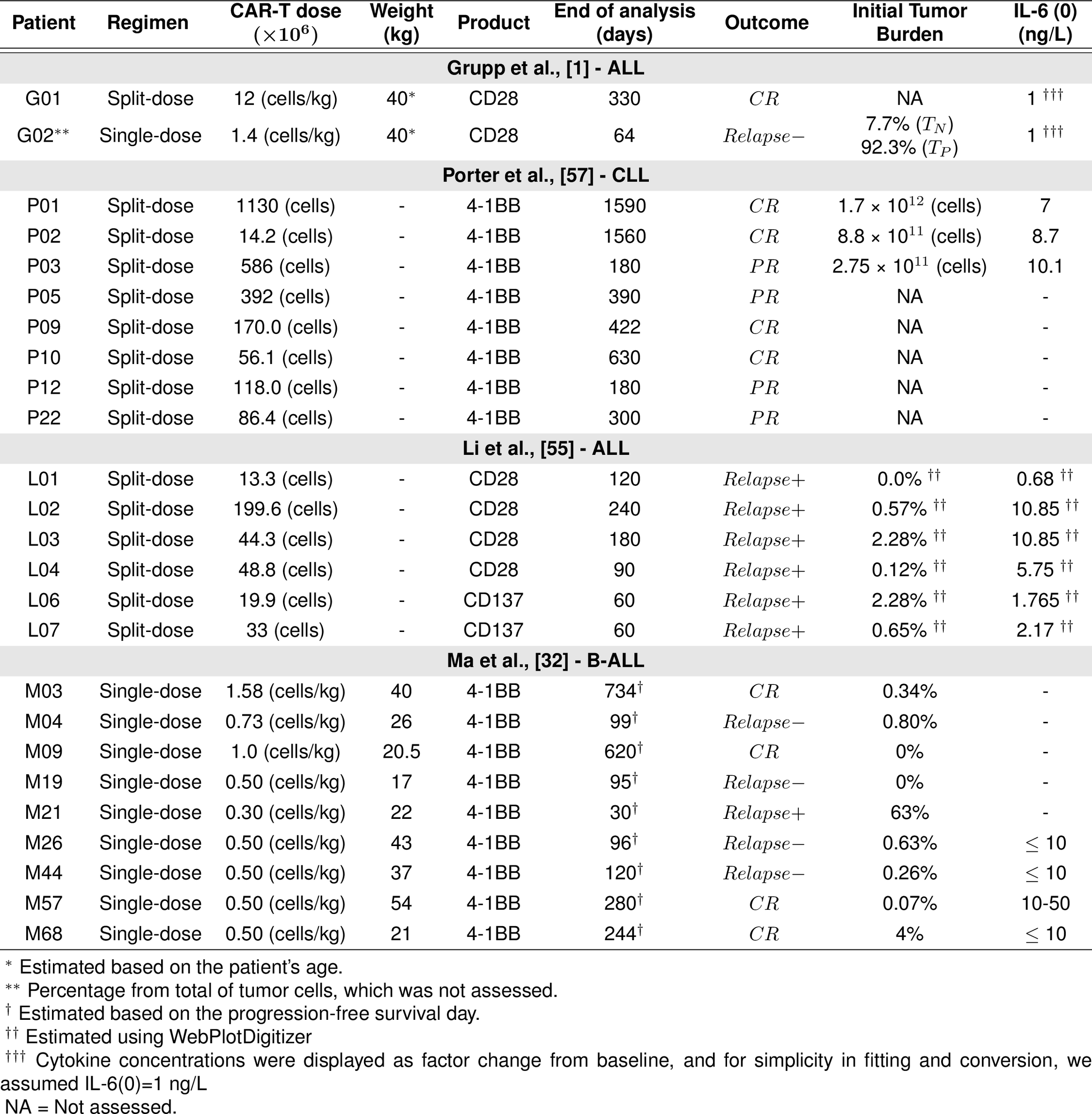

Supplement: S1 Table — Individual CAR-T cell dose, the end of analysis, disease, outcome, initial tumor burden and baseline IL-6 concentration. In the split-dose regimen, the CAR-T cell dose was given in 3 fractions with 10% administered on day 0, 30% on day 1, and the remaining 60% on day 3. (TIF) [file pcbi.1012908.s013.tif]

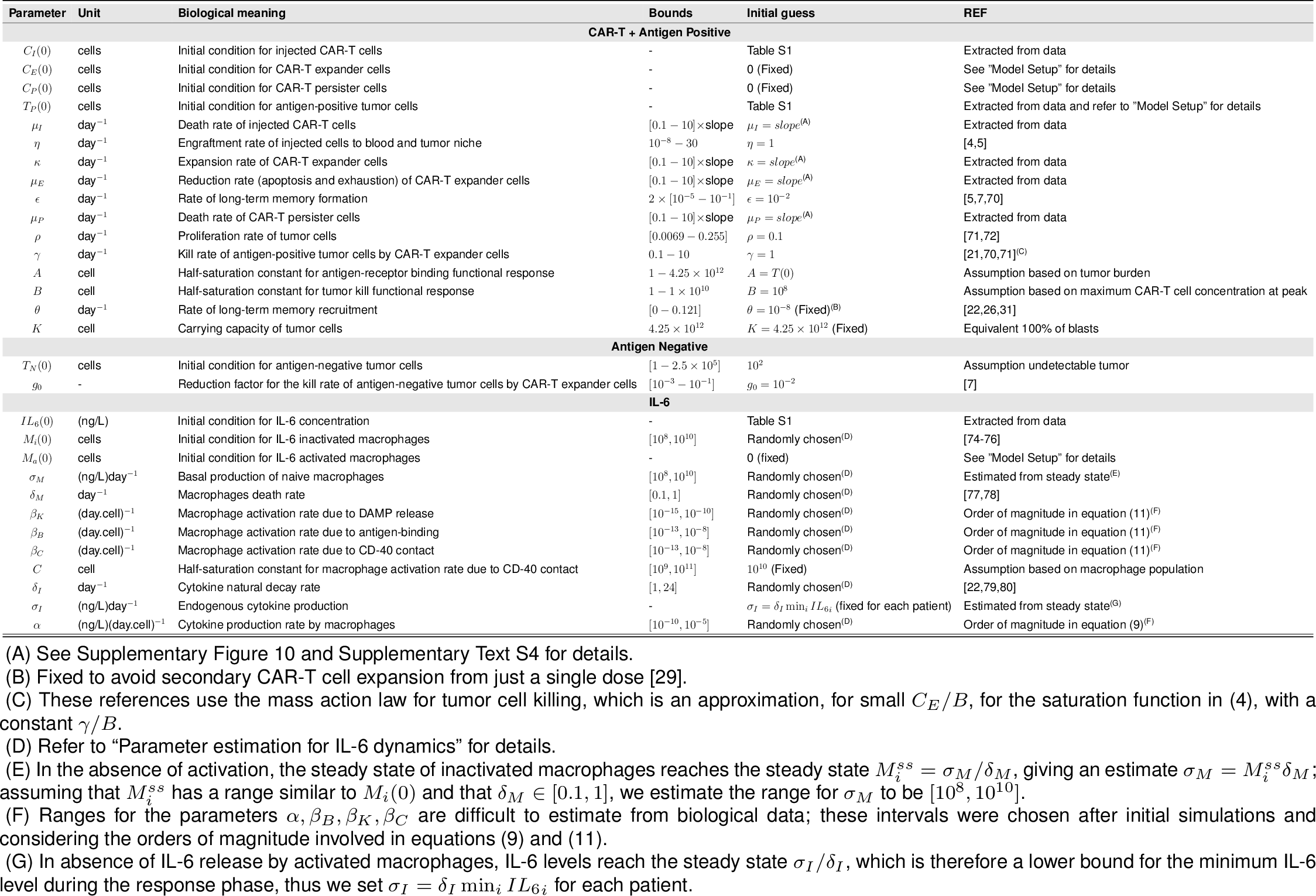

Supplement: S2 Table — Model parameters, their biological meanings, bounds, and references used for parameter estimation. (TIF) [file pcbi.1012908.s014.tif]

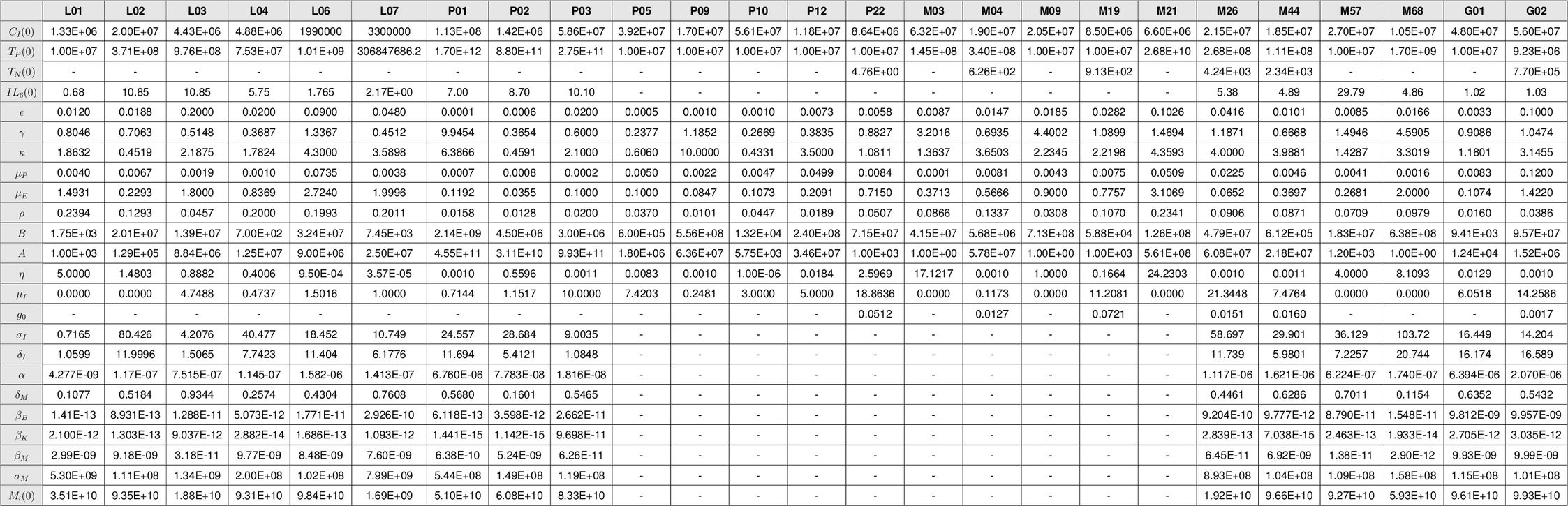

Supplement: S3 Table — Patient-specific fitted parameters. Parameters whose values were the same for all patients are: CT(0)=CP(0)=Ma(0)=0,K = 4 . 25 × 1012 cells and θ=10−8 day−1. (TIF) [file pcbi.1012908.s015.tif]
