# Supplementary material for: Mathematical modeling unveils the timeline of CAR-T cell therapy and macrophage-mediated cytokine release syndrome
Source: PLoS Comput Biol. 2025 Apr 9;21(4):e1012908. doi: 10.1371/journal.pcbi.1012908 (PMC11981663; doi:10.1371/journal.pcbi.1012908)
Supplement: Attachment — Submitted filename: Review.pdf [file pcbi.1012908.s016.pdf]

In this manuscript, the authors present a compartment model with CAR-T cells, tumour cells and microphages to understand the cell dynamics in CAR-T cell therapy and CRS. Extending from their previous work of CAR-T therapy, they focus on understanding the role of three different macrophage activation ways on CRS by fitting patient data fitting with their model. The model fitting is done first at the CAR-T and tumour cell part and then at the macrophages part, as the two dynamics are independent in the model set up. Both CAR-T and IL-6 data have a pattern of increasing first and decreasing later in terms of abundance for all patients, which agrees with the components of the model setup. By fitting the IL-6 data with different combinations in the macrophage activation ways, the authors concluded that in the function of macrophage activation rate, the term modelling interactions between activated macrophage and CAR-T expanders are needed for the best data fitting.

While I think the current deterministic model can not really capture the tumour dynamics, it does reflect the dynamics of CAR-T and IL-6. Thus, I suggest a revision with following suggestions:

Major points:

1. The mathematical modelling part is straight forward, but lengthy and wordy. I would suggest to put the rates in equations 1) to 10) in proper positions in Figure 1 to improve the readability.

2. I appreciate the authors tried to list the source of picking up specific parameter values. However, this is a multi-compartment model with many parameters. Realisations of specific parameters are snapshots of full dynamics. While each parameter might have specific values in certain literatures (and some citations are theoretical models as well), it is hard to say whether they holds together in the same real system. Instead, the power of computational work is that we can have basic analysis to understand the impact of the parameters.

I would suggest beside of data fitting, the authors also include some figures systematically explaining the impact of basic components in the dynamics. For example, as η, κ, ϵ, θ are coefficients for the transition between different CAR-T cell compartments, whether and how the order of those parameters impact on the outcomes of tumour progress and treatment response? Whether and why it

is important to have different death rates (μ) for CAR-T cells (C_I, C_E and C_P), but the same death rates for the different macrophages (M_i and M_a)? What is the importance of having K, the carrying capacity in the tumour population? I noticed that in Figure 4, you have some discussion on this direction but very descriptive. I would suggest to have, for example, a systematic figure on this.

3. In Figure 2, the fitting of the CAR-T cells and IL-6 seem to be fine. This is expected as, in the model setup CAR-T cells and IL-6 have relatively independent dynamics. With the increase, decrease and decay terms in CAR-T and activated macrophage equations, the fitting should work in some parameter fitting.

However, in the same figure we can see the fitting does not work well for the tumour cells. The authors should give a proper discussion on this in the results.

4. Figure 3, explain in the text why sometimes is min slope and sometimes is maxi slope in the x-axis of figure 2 panel and how do you calculate them from the data? In addition, in Figure 3b-h, x- and y-axis are all fitted from the data which should be correlated to each other, thus I am bit confused by the message you want to deliver behind these figures?

5. Please correct the notation of C throughout the manuscript. For example, C is both the total CAR-T cells (e.g. Line 631), the saturation parameter in eq. 11) and the index of j for CD40 in eq. 13).

6 In figure 5 caption, the authors wrote ”e. however, for the majority of patients, the model predicts that ...” Are the dashed lines the best linear fit of the estimated x-y values of patient data? If just looked by eye, figure 5e could also be two-clusters in the x-axis (time of LI-6 peak).

Minor points:
In line 553, $C_I(t)$ instead of $C_D(t)$?
in Line 575, ? = 0.
In line 586, a minus is missed in eq. 7).
