# Supplementary material for: Mathematical modeling unveils the timeline of CAR-T cell therapy and macrophage-mediated cytokine release syndrome
Source: PLoS Comput Biol. 2025 Apr 9;21(4):e1012908. doi: 10.1371/journal.pcbi.1012908 (PMC11981663; doi:10.1371/journal.pcbi.1012908)
Supplement: Attachment — Submitted filename: Response to Reviewers.pdf [file pcbi.1012908.s017.pdf]

Response to Reviewers

PCOMPBIOL-D-24-01476

Mathematical modeling unveils the timeline of CAR-T cell therapy and macrophage-mediated cytokine release syndrome

Reviewer #1:

In this manuscript, the authors propose a new mathematical model of CAR-T cell cancer therapy that includes CAR-T cell dynamics of expansion and memory formation, antigen-negative resistance, and macrophage-associated cytokine release. Cytokine release syndrome (CRS) can be a severe side-effect of CAR-T cell therapy. The model was calibrated on a dataset of 25 patients collected from published literature.

The model was built on three different layers: CAR-T cell dynamics, tumor cell dynamics, and macrophage dynamics. The model consists of eight ordinary differential equations describing CAR-T cell subsets (three equations), tumor subpopulations (two equations), macrophage subsets (two equations) and IL-6 release (one equation). Model inference was made possible by fitting parameter layer-wise, first CAR-T cell and tumor dynamics, then macrophage activation and IL-6 release.

The manuscript focuses on the mechanisms leading to macrophage activation and the ensuing IL-6 release following CAR-T injection. Three macrophage activation mechanisms were investigated: DAMPs release (molecules released by dying tumor cells), CAR-T cell antigen recognition, and cell-cell contact though CD40-CD40L interaction. Using IL-6 release as a proxy for CRS, the contribution of each mechanism to CRS was quantified. CD40/CD40L interaction was identified as the most important factor. Mitigation strategies were searched by sensitivity analysis. CAR-T cell doses, or preconditioning treatments that reduce initial tumor burden could decrease the risk of severe CRS. Pharmaceutical inhibition of macrophage activation soon after CAR-T infusion could also prevent cytokine release.

The manuscript presents several interesting results. First, the collected dataset is by itself of interest, and the authors show that primary clinical parameters are not necessarily good predictors of IL-6 release. Second, by decomposing the contribution of each macrophage activation mechanism, the authors show that the mechanisms are temporally distinct. This in turn is helpful for setting up strategies for limiting cytokine release syndromes. Overall, this study shows how combining data analysis and mathematical modeling can be used to propose better treatment combinations/protocols for mitigating severe side effects of CAR-T therapies.

Thank you for the comprehensive summary of our work. We are pleased that the reviewer appreciates the novelty of our model, the value of the dataset we assembled, and the implications of our findings. Below, we provide a point-by-point response to each specific comment, along with details of the corresponding revisions made to the manuscript. Line numbers indicated in our responses correspond to the manuscript with tracked changes.

Comments

1 - The weakest link of this study is the assumption that IL-6 release is a strong indicator of severe CRS. The authors fail to directly address this assumption, except in passing (lines 105-107, 111-114). Yet, one of the main conclusion is that ... our modular model structure accurately describes ... the occurrence of macrophage-mediated CRS. (lines 125-128). The link between IL-6 and the risks of severe CRS should be discussed and backed-up more clearly.

Thank you for this objection. We agree that further clarification and support are needed for the assumption that IL-6 is a strong indicator of severe CRS. In our study, IL-6 was chosen as a representative biomarker for CRS due to its well-established central role in the pathophysiology of CRS. For example, a recent review (PMC8600921) emphasizes: *"Among the cytokines released by monocytes/macrophages, including IL-6, IL-1, IL-2, TNF-α, GM-CSF, and IFN-γ, IL-6 plays a central role"*. Morris et al. (Nat. Rev. Immunol. 10.1038/s41577-021-00547-6) also state: *"The empirical testing of different blocking antibodies soon identified IL-6 as a crucial mediator of CRS."* They further note that "*IL-6 blockade can lead to the reversal of most symptoms and across-the-board cytokine downregulation in many patients. In preclinical models, IL-6 was also found to mediate mortality from CRS*" indicating the centrality of IL-6 in CRS pathophysiology and severity.

However, we are aware of the multifactorial nature of CRS, and the limitations of solely using IL-6 as a surrogate marker for CRS. Therefore, and to address your concern, we replaced several instances of "CRS" with "cytokine release" or "IL-6 release" to avoid overgeneralizing the relationship between IL-6 and CRS severity. We have also refined some conclusions to ensure they better reflect the scope of our study (section starting at line 347). Also, a discussion paragraph was included to explicitly acknowledge the limitations of using IL-6 as a sole indicator of CRS severity (lines 565-575). While IL-6 may be a surrogate marker for CRS, we highlight the multifactorial nature of CRS and discuss that our modular framework can accommodate additional cytokines or biomarkers in the future.

2 - The sensitivity analysis is local only (one parameter at a time). Did the authors consider performing a global sensitivity analysis (Sobol sensitivity analysis, Fourier amplitude sensitivity test…) to get a broader quantification of sensitivity outside the best-fit parameter values.

Thank you for raising this interesting point. We agree with the reviewer about the relevance of global sensitivity analysis in some contexts. However, we believe that the nature of our approach and the specific goals of our study do not call for such an analysis.

Indeed, our approach is distinct in that we derive biologically interpretable parameter values directly from patient data. Specifically, for the first layer of the model (governing CAR-T and tumor cell dynamics), we are able to map initial guesses for 10 out of 12 parameters using direct features of the patient-specific data. This is made possible by the insights given by our time-scale analysis, which link each phase of the multiphasic dynamics (described by slopes in the log-scale) to a corresponding mechanistic parameter driving that phase. From this analysis, we develop an approach where the observed slope values from patient data

provide a direct estimate for these mechanistic parameters, ensuring that the best-fit values are biologically grounded and interpretable.

This biologically-informed approach contrasts with global optimization techniques, where parameter sets are explored without specific guidance from the data structure. In our case, a global sensitivity analysis may not add meaningful insights, as our parameter estimation process already inherently ties the dynamics to individual parameters through a mechanistic and interpretable framework.

We acknowledge that this rationale may not have been sufficiently clear in the original manuscript. To address this, we have revised the text (lines 170-174 and section starting at line 180) to better emphasize this contribution, which we view as a major strength of our work: the ability of a minimal model to map observable features of CAR-T cell therapy responses (phenomenological parameters) onto mechanistic model parameters. Following a suggestion of Reviewer #2, we have also included Supplementary Fig. S5, which systematically analyzes the impact of each basic parameter in the dynamics of CAR-T and tumor cells, and was the basis for Fig. 4.

For the third layer of the model, we performed a local sensitivity analysis (Fig S10) by varying the parameters by ±50%. This analysis demonstrates the robustness of our conclusions, particularly the role of CD40 contact as the main mechanism driving macrophage recruitment and subsequent IL-6 release.

Lines 173, 181: parameters should be described when first introduced in the text.

Thanks. We have included sentences that describe the parameters (lines 187, 195).

Line 177: ‘such as’ should be removed, the description lists all four parameters.

Thanks. We have replaced “such as” by “namely (line 192)”.

Line 182: Does the sensitivity analysis concern all parameters or only a subset? Please clarify.

Thanks for pointing this out. The sensitivity analysis concerns 10 out of 12 parameters. We have included the details (section starting at line 180, line 199).

Lines 187-190: As they are reported, it looks like all measured responses depend on all parameters. Perhaps put the results into perspective by indicating also which response/parameter pairs are independent?

We acknowledge the inconsistency and thank the reviewer for drawing our attention to this point. Although the pair (Cmax,Tmin) depends on six parameters (\kappa, \rho, \gamma, \mu_E, A and B), we now emphasize that the first four are initially fixed by other characteristic slopes. Therefore, only the pair (A,B) can be varied to match the pair (Cmax,Tmin). We have incorporated this reasoning (lines 209-216).

Section starting on line 216: As far as I can see, this section is devoted to data analysis only, and is independent from the mathematical model. It is unclear why modeling is referred to (on lines 222-223).

In this section, we analyze the correlations between some of the measures obtained from the model fits. We believe this approach is better because the model simulations provide additional measures that are not always available from the data, such as time of macrophage peak, tumor load at different time points. We acknowledge that the previous wording was not clear. We reworded this section in several aspects (lines 247-249, other instances in that section).

In the same section, correlation results are not supported by statistical analysis. Basic regression analysis should be provided, along with relevant statistics (n, p-values, R^2, F values).

Thank you for your suggestion. In response, we have included the best linear fits, along with the corresponding R2 and p-values, in all panels of Figure 5. Additionally, in panels (e) and (f), the previously shown dashed lines represent the range where the CAR-T cell and IL-6 peaks differ by less than 5 days (and macrophage and IL-6 peaks differ by less than 2 days). To avoid confusion with the best linear fits, we have replaced these dashed lines with a shaded region.

Moreover, on lines 231-234, conclusions are drawn on CRS, no data on CRS is provided.

We have reformulated the conclusions, now reading as "...at the patient level, the increase in IL-6 levels and its likely association with CRS does not depend solely on any of the above factors...". We also adjusted the conclusion about anticipating CRS (now IL-6 peak) by monitoring macrophage levels. Please see also our response to first comment above (lines 275, 292, several instances in that section)

Line 235: 'unveiled'. Is this analysis original? If so, it should be emphasized, or put into perspective with related clinical observations.

Thank you for the detailed reading. Indeed, our results are not the first to report this connection. We have replaced "unveiled" by "confirmed" to avoid overstatement (line 269).

Line 245. 'Neglecting the patients who achieved sustained remission…' This clearly poses statistical difficulties. Some of the strong responders have low CAR-T fold changes, so it is not clear why these patients can be excluded.

Thank you for pointing this out. Initially, we excluded patients with simulated zero tumor cells at days 28 or 90 from the analysis to avoid computational issues with the log-log scale. However, we recognize that this approach could introduce statistical bias. We have recalculated the analysis, now including all patients (a value of 0.1 cells is assigned for cases where the model predicts zero tumor cells). Although some correlation coefficients change slightly, the overall conclusions remain unchanged: a weak correlation is observed between CAR-T fold change and tumor response at day 28, and a moderate correlation persists between tumor responses at days 28 and 90 (lines 279-290 and caption of Fig 5).

Line 271-273: 'This suggests that smaller CAR-T cell doses…' It is not immediately clear if the modeling results are being interpreted in real clinical setting, i.e. that 'smaller CAR-T cell doses extend the expansion phase…' is a model prediction, or if the extension of the

expansion phase is the proposed interpretation of the observation that ‘smaller doses resulted in decreased IL-6 fold change and delayed CAR-T cell and IL-6 peaks’.

Thank you for pointing this out. The model predicts that using smaller doses results in decreased IL-6 fold change and delayed CAR-T cell and IL-6 peaks, without changing the CAR-T cell peak height (Cmax). Based on these predictions, our interpretation is that smaller CAR-T cell doses extend the expansion phase. We have reformulated the paragraph to clarify this distinction and included the wording “simulating/simulated” in several instances of this section to achieve a better consistency (section starting at line 295, lines 303, 307, etc).

Line 287: ‘It rather appears that a saturation arises…’ Please clarify what is meant by ‘saturation’; I don’t see it from Fig 6d alone.

Thank you for your feedback. By “saturation” we refer to the observation that while IL-6 peak levels increase with higher initial tumor burden or CAR-T cell dose, the magnitude of this increase becomes smaller as the simulated values rise (e.g., when tumor burden is increased by 10x and 100x, as shown in Fig. 6d). To clarify this aspect, we have revised the paragraph and now explicitly describe this behavior and better explain our interpretation (lines 327-334).

Lines 293-295: The sentence seems redundant here, as the results are reported two paragraphs above.

Thank you for this observation. We agree and have merged the content with the previous paragraphs (lines 336-341)

Line 301-302 ‘CRS timeline’ could be replaced by ‘IL-6 release/fold change timeline’, unless compelling evidence that CRS = IL-6 fold change.

Thank you. In line with the answer to the first point above, we changed “CRS timeline” to “IL-6 release”. We have also revised several instances in the manuscript to make it consistent.

Line 312: ‘beta_B, beta_C = 0’ -> ‘beta_B = 0, beta_C = 0’. Similar comments for lines 313, 315 and 318.

Thank you for your careful reading. We have modified the manuscript accordingly (lines 358, 360, 362, 365).

Line 373: Wang et al (2022) https://doi.org/10.3389/fimmu.2022.814548 state that severe CRS appear in average two days post-CAR-T cell infusion (earlier that mild CRS). How does the proposal that blocking macrophage activation 1-3 days post-infusion compare with these observations? More generally, if CRS appear soon after infusion, then CD40-mediated macrophage activation, which occurs late, would not be the main driver for CRS. This should be discussed.

Thank you for this helpful comment and for pointing out this reference. In Fig 8b, the results suggest that initiating the blockade between day 0 up to to day 3 post-infusion, is, on average, the most effective strategy for mitigating macrophage activation. For certain patients, the intervention should occur even earlier (e.g., within 2 days, note the gray lines increasing from day 2). This was not fully clear in the original manuscript, where we

incorrectly stated that “blocking macrophage activation 1 or 3 days post-infusion is most effective.” We have corrected this to reflect the actual range (0 to 3 days, line 420).

We also acknowledge the multifactorial nature of CRS and the possibility that other pathways may drive early severe CRS in some patients. For example, our model predicts that in cases such as patients L3 and P03 (Figure 7b), DAMPs release - the earliest mechanisms triggered post-infusion - may be the primary driver of macrophage activation. We also note that the median time to CRS onset of 2 days (range 0 to 5) reported by Wang et al (2022) refers specifically to the severe CRS group (n=7 patients), whereas the mild CRS group (n=47 patients) had a median onset of 7 days (range: 0 to 13). In our view, this is aligned with our interpretation, suggesting that additional pathways (e.g., DAMPs or other mechanisms) may be involved as major drivers in the rarer cases of early and severe CRS.

We have revised the manuscript to clarify the timing of macrophage activation blockade in Fig 8b, and discussed the potential role of alternative mechanisms in early and severe CRS (Discussion section, lines 502-507).

Lines 454-479 discusses the framework used for CAR-T cell activation paths. While this discussion is an interesting topic, it is not central to this manuscript. Unless the modeling choices for CAR-T cell fate decision are important for the results (I guess the author assume that their results are relatively robust), this section could be shortened.

Thank you for your suggestion. We agree that this discussion can be condensed ande revised the paragraph accordingly (lines 508-542).

Equations

I have not checked the equations thouroughly, but the simulation results seem in agreement with the model description.

Thanks.

We thank the reviewer for all the comments and suggestions, which improved the quality of our manuscript.

Reviewer #2

In this manuscript, the authors present a compartment model with CAR-T cells, tumour cells and microphages to understand the cell dynamics in CAR-T cell therapy and CRS. Extending from their previous work of CAR-T therapy, they focus on understanding the role of three different macrophage activation ways on CRS by fitting patient data fitting with their model. The model fitting is done first at the CAR-T and tumour cell part and then at the macrophages part, as the two dynamics are independent in the model set up. Both CAR-T and IL-6 data have a pattern of increasing first and decreasing later in terms of abundance for all patients, which agrees with the components of the model setup. By fitting the IL6 data with different combinations in the macrophage activation ways, the authors concluded that in the function of macrophage activation rate, the term modelling interactions between activated macrophage and CAR-T expanders are needed for the best data fitting.

While I think the current deterministic model can not really capture the tumour dynamics, it does reflect the dynamics of CAR-T and IL-6. Thus, I suggest a revision with following suggestions:

Thank you for your comprehensive reading of our manuscript. We acknowledge that our model is more effective in capturing the dynamics of CAR-T cells and IL-6, as these are supported by richer datasets. However, we believe that our model also captures the qualitative behavior of tumor dynamics, despite the inherent limitations posed by the sparse data points available for tumor cells. We appreciate the opportunity to clarify this aspect and provide further details in our responses to your specific comments below. Line numbers indicated in our responses correspond to the manuscript with tracked changes.

Major points:

1. The mathematical modelling part is straight forward, but lengthy and wordy. I would suggest to put the rates in equations 1) to 10) in proper positions in Figure 1 to improve the readability.

Thank you for your critical reading and suggestion. We have added the rates to the appropriate positions in Figure 1, and we agree that this change improves the overall readability of the manuscript. Given the online format and lack of space restrictions in PLOS Computational Biology, we aimed to provide detailed information on the modeling process, assumptions, and data curation steps to ensure both clarity and reproducibility. However, in response to your feedback, we have condensed the modeling section by removing and shortening several aspects without compromising clarity (lines 625-692).

2. I appreciate the authors tried to list the source of picking up specific parameter values. However, this is a multi-compartment model with many parameters. Realisations of specific parameters are snapshots of full dynamics. While each parameter might have specific values in certain literatures (and some citations are theoretical models as well), it is hard to say whether they holds together in the same real system. Instead, the power of computational work is that we can have basic analysis to understand the impact of the parameters.

I would suggest beside of data fitting, the authors also include some figures systematically explaining the impact of basic components in the dynamics. For example, as η, κ, ϵ, θ are coefficients for the transition between different CAR-T cell compartments, whether and how the order of those parameters impact on the outcomes of tumour progress and treatment response? Whether and why it is important to have different death rates (μ) for CAR-T cells (CI, CE and CP), but the same death rates for the different macrophages (Mi and Ma)? What is the importance of having K, the carrying capacity in the tumour population? I noticed that in Figure 4, you have some discussion on this direction but very descriptive. I would suggest to have, for example, a systematic figure on this.

Thank you for raising these important points. We completely agree that parameter realizations are snapshots of the full parameter space and that systematically analyzing the impact of parameters is crucial to understanding the dynamics of the model. While we recognize the value of this perspective, we believe that many of the answers to these questions are already embedded in our approach, although we acknowledge that they were not clearly explained in the original manuscript.

Specifically, before running the parameter estimation procedure, we conducted an analytical exploration of the model's time scales. This analysis identified six distinct dynamic phases in the CAR-T and tumor cell dynamics (four for CAR-T cells and two for tumor cells), each predominantly driven by specific model parameters, including the death rates (μI,μE,μP), the activation rate (κ), and the tumor growth and killing rates (ρ and γ). This allowed us to directly estimate these six parameters from individual slopes read from the patient time-series data. In addition, our sensitivity analysis (Figs 4, S5, S6) revealed that the model behavior with respect to four other parameters (η,ϵ,A,B) is intrinsically related to four other key features in the patient data, namely the minimum and peak values of the CAR-T and tumor cells, which can also be estimated.

This stepwise approach ties our locally optimized parameters directly to biologically interpretable phenomenological parameters embedded in the patient data, ensuring that the parameter values are consistent with both the data and biological plausibility. For example, the lower death rate (μP) for persister CAR T cells is consistent with their long-lived phenotype.

Regarding the carrying capacity of tumor cells (K), our analysis shows that this parameter is relatively insensitive. Assuming exponential tumor growth would yield similar results, but we retained the carrying capacity for biological plausibility. Concerning the macrophage death rates, given the absence of macrophage time-course data, we assumed equal death rates for activated and inactivated macrophages to maintain parsimony.

We acknowledge that our original manuscript did not sufficiently emphasize these aspects or the systematic nature of our sensitivity analysis. Thus, we have made several revisions: i) A new Supplementary Figure has been added (Fig. S5), which was the basis for Fig. 4, where we systematically examine the effect of each of the 12 parameters in the basic model on CAR-T and tumor cell dynamics. ii) In lines 170-174, and in the section starting at line 180, we have clarified the strengths of our approach and its biological interpretability, and provided a more detailed explanation of our sensitivity analysis, explicitly mentioning the

insensitive parameters (lines 201-204). In the Methods section, we have added more information about the choice of certain parameters, too.

3. In Figure 2, the fitting of the CAR-T cells and IL-6 seem to be fine. This is expected as, in the model setup CAR-T cells and IL-6 have relatively independent dynamics. With the increase, decrease and decay terms in CAR-T and activated macrophage equations, the fitting should work in some parameter fitting.

However, in the same figure we can see the fitting does not work well for the tumour cells. The authors should give a proper discussion on this in the results.

Thank you for this comment. We appreciate the opportunity to clarify and discuss this point. We agree that the tumor cell dynamics are not well captured in Figure 2 for **patient L02**, where our model successfully reflects the relapse around day 200 but did not predict the earlier relapse at day 120. For **patient L04**, the model accurately captured the observed relapse. For **patients G02 and M44**, we did not have access to direct tumor burden measurements at the relapse points. Instead, the available data provided only qualitative information about the timing of a relapse of antigen-negative tumor cells. In these cases, the red curve in the previous version of Figure 2 describes the decrease of antigen-positive tumor cells, while the blue curve represented the dynamics of antigen-negative tumor cells, capturing the relapse. To avoid misinterpretation, we have updated the figure by changing the color of the antigen-negative tumor cells, which are now shown as red dashed lines. For the other four patients in Figure 2, the dataset included only the initial tumor burden and confirmation that no relapse occurred within specified time points. In **Figure S2**, the model correctly fits the relapse of antigen-positive tumor cells for all seven patients. Similarly, in **Figure S3**, the model captures antigen-negative tumor relapse for six patients.

We recognize that the fitting of tumor dynamics is limited by the sparsity and qualitative nature of some tumor cell time-courses. However, this limitation does not invalidate our approach. We have expanded the discussion (lines 549-556) to emphasize the importance of more refined and frequent tumor cell measurements to improve the predictive power of future models. While we acknowledge that such data collection is not straightforward for all tumor types, it remains a critical goal for advancing the field.

4. Figure 3, explain in the text why sometimes is min slope and sometimes is maxi slope in the x-axis of figure 2 panel and how do you calculate them from the data? In addition, in Figure 3b-h, x- and y-axis are all fitted from the data which should be correlated to each other, thus I am bit confused by the message you want to deliver behind these figures?

We compare the maximum and minimum slopes of the model solution fits during each phase with the parameter values identified by analytical approximations as the primary drivers of that phase. The relevance of the maximum or minimum slope depends on its association with the parameter in question. In Figure 3b-h, the x-axes represent the slopes (mD, mE, mC, mP, mT, mS, mR) derived from the model-fitted solutions, while the y-axes show the best-fit values of the corresponding mechanistic parameters (growth/decay rates \mu_i, k-\mu_E, \rho, \gamma, \rho-\gamma). The observed correlation confirms that our analytical

approximations successfully identify the parameter governing each phase. This supports our stepwise parameter estimation approach in which the slopes obtained from segmenting the patient time courses (Fig S11) are used to inform the values of the corresponding mechanistic parameters. This biologically informed estimation is further refined in the subsequent section. We have added several clarifications in the text (section starting at line 131).

5. Please correct the notation of C throughout the manuscript. For example, C is both the total CAR-T cells (e.g. Line 631), the saturation parameter in eq. 11) and the index of j for CD40 in eq. 13).

Thanks for pointing this out. We have replaced the total number of CAR T cells with C_T (lines 718-733), and the indexes j=K,B,C in the section starting at line 853 with j=1,2,3, thus removing the ambiguity in the notation of C.

6. In figure 5 caption, the authors wrote ”e. however, for the majority of patients, the model predicts that ...” Are the dashed lines the best linear fit of the estimated x-y values of patient data? If just looked by eye, figure 5e could also be two clusters in the x-axis (time of LI-6 peak).

In this figure, the dashed lines represent the range where the CAR-T cells and IL-6 peaks differ in less than 5 days. Thus, our message is that the majority of patients fall in this region, thereby indicating the strong temporal association. We have replaced the two dashed lines with a shaded region, in order to avoid confusion with best linear fits. On the other hand, following the suggestion of reviewer 1, we have also included the best linear regression (along with R2 and p-values in all panels of Figure 5).

Minor points:

In line 553, CI(t) instead of CD(t)?

Thank you for your careful reading. We have corrected this typo (line 632).

in Line 575, ? = 0.

The condition \vartheta=0 refers to a parameter of our previous model, where now we removed this parameter. Following your comment #1, we have shortened the modeling part, removing this confusing detail (line 657).

In line 586, a minus is missed in eq. 7).

Thank you for your careful reading. We have corrected this typo (line 668).
