# Supplementary material for: Mathematical modeling unveils the timeline of CAR-T cell therapy and macrophage-mediated cytokine release syndrome
Source: PLoS Comput Biol. 2025 Apr 9;21(4):e1012908. doi: 10.1371/journal.pcbi.1012908 (PMC11981663; doi:10.1371/journal.pcbi.1012908)
Supplement: Attachment — Submitted filename: RevisonReview.pdf [file pcbi.1012908.s018.pdf]

The authors have addressed most of my comments except the following one.

I appreciate in the response the authors honestly acknowledged the sparsity of the tumour data, which is understandable and unfortunately makes it hard to fully access the accuracy of model prediction (i.e. for the four patients, 2 patients have no relapse data to compare with the model. The other two patient, one has two data points, one has three data point, where the two data points one worked well.) I originally suggested to have a discussion on this point and allow the audience to be aware of this while applying this model.

In their response letter, the authors wrote ”We agree that the tumor cell dynamics are not well captured in Figure 2 for patient L02,where our model successfully reflects the relapse around day 200 but did not predict the earlier relapse at day 120. For patient L04, the model accurately captured the observed relapse. For patients G02 and M44, we did not have access to direct tumor burden measurements at the relapse points....We recognize that the fitting of tumor dynamics is limited by the sparsity and qualitative nature of some tumor cell time-courses. However, this limitation does not invalidate our approach. We have expanded the discussion (lines 549-556) to emphasize the importance of more refined and frequent tumor cell measurements to improve the predictive power of future models.”

Unfortunately, if the line numbers listed are correct, I failed to see a clear statement in line 549-556 but rather some treatment details of each patient.
